# Editorial: eDiagnostics and monitoring for precision endocrinology

**DOI:** 10.3389/fendo.2023.1229475

**Published:** 2023-07-04

**Authors:** Simmi Kharb, Anagha Joshi

**Affiliations:** ^1^ Department of Biochemistry, Postgraduate Institute of Medical Sciences, Rohtak, Haryana, India; ^2^ Computational Biology Unit (CBU), Department of Clinical Science, University of Bergen, Bergen, Norway

**Keywords:** omics, eDiagnostic tools, exposome, external factors, diagnosis, prognosis and treatment

Artificial intelligence and machine learning, personalized data management, clinical and epidemiological data, computational resources, preterm birth and other pregnancy-related and endocrinal pathologies, genomics, pharmacogenetics, e-monitoring and interdisciplinary approaches, precision medicine and personalized therapies.

The precision endocrinology monitoring is yet to be established in routine clinical practice, providing medical professionals and patients with the tools for a better understanding of the complexities of therapy and appropriate management of various endocrine disorders. The goal of this series is to investigate the role of precision medicine in the precise biochemical diagnosis of endocrine disorders, the appropriate use of genetic analysis, and their incorporation into translational treatment for these disorders. To develop acceptable and affordable interventions for eDiagnostics and precision monitoring in the real world, it is necessary to integrate sensor data obtained from electronic medical records, medical, demographic, and social variables using advanced machine learning tools.

Pregnancy is a stage in female cycle with drastic hormonal changes and its effects on short (perinatal complications) and long term (e.g. cancer risk) female health including cancer risk. Prediction of adverse pregnancy outcomes would allow women at high risk to get therapeutic and prophylactic interventions while other being spared from needless care. This includes developing methodologies to treat at-risk women during and after pregnancy by understanding the interplay between molecular, genetic, and clinical factors related to adverse pregnancy outcomes and accurately assessing the risk of adverse pregnancy outcomes well in advance of their occurrence. National efforts such as “Nulliparous Pregnancy Outcomes Study: Monitoring Mothers-to-Be” study allow active data collection for risk assessment and model development by close partnership between computational and computational and clinical scientists to gain deeper understanding of adverse pregnancy outcomes by development of machine learning models for advanced risk prediction.

Females are more likely to suffer from autoimmune diseases such as Systemic lupus erythematosus (SLE). In patients with SLE, adverse pregnancy outcome occurs in about 20% of pregnancies, namely, preeclampsia (PE), abortion, preterm birth, stillbirth, renal failure and fetal growth restriction. The machine learning (ML) methodology (1) has been applied to a large cohort to identify additional predictors of adverse pregnancy outcome with mild to moderate SLE both with and without antiphospholipid antibody positivity. In this series, the first article by Deng et al. addresses the potential genetic biomarkers to predict adverse pregnancy outcomes during early and mid-pregnancy in women with systemic lupus erythematosus. They identified three predictive gene biomarkers (SEZ6, NRAD1, and LPAR4) of adverse pregnancy outcome with SLE. These findings would improve the understanding of the pathogenesis of adverse pregnancy outcome in women with SLE and contribute toward the development of personalized clinical management of pregnant women with SLE. Other machine learning strategies have also shown a promise such as EUREKA algorithm predicted obstetric risk in patients with different subsets of antiphospholipid antibodies (2). The utility of ML in aiding clinical risk stratification requires further validation and similar methodological approaches could be trialed across the autoimmune connective tissue disease spectrum to provide better prognostic information to patients at diagnosis, irrespective of their diagnostic label. Furthermore, infertility or subfertility is common in patients with autoimmune diseases (3). The study by Chen et al. reported the effects of assisted reproductive technology on gene expression in the heart and spleen tissues of adult offspring mouse. In the mouse model, ART can interfere with the gene expression pattern in the heart and spleen of the adult offspring and these changes are related to the aberrant expression of epigenetic regulators. Previous models of risk score incorporating social determinants along with clinical and demographic factors in at-risk populations have been reported to have limited performance and future research may focus on the development and validation of more sensitive risk tools for the use impact of implementing risk tools in the setting of pregnancy case management programs.

Innovative genomic technologies offer unlimited possibilities including the development of individualized care, based on unique genomic information. The next article by Li et al. reported on integrated analysis of fibroblast molecular features in papillary thyroid cancer combining single-cell and bulk RNA sequencing technology. Papillary thyroid cancer (PTC) is the most common thyroid cancer. In this study, single-cell sequencing data of PTC were analyzed and demonstrated fibroblast features in the thyroid cancer microenvironment which may provide potential value for thyroid cancer treatment in future. Zhang, Wang, X.-N. et al. presented a systemic review and meta-analysis on conventional ultrasonography and elastosonography in the diagnosis of malignant thyroid nodules. The existing evidence indicates that elastosonography cannot completely replace conventional ultrasound in the diagnosis of malignant thyroid nodules, and the combination of elastosonography and conventional ultrasound gives better diagnostic precision. Precision medicine in future would facilitate more accurate diagnosis and therapy at the intrinsic molecular level, allowing the customization of healthcare and incorporation of electronic medical information obtained both on an individual and on a global scale. Another research article by Zhang, Wang, C. et al. reported robotic bilateral axillo-breast versus endoscopic bilateral areola thyroidectomy outcomes of 757 patients. In their study, they documented that the bilateral axillo-breast approach (RT-BABA) is as safe and feasible as ET-BAA and even performed better in some surgical outcomes. Further prospective studies to confirm the safety of RT-BABA are needed.

The final review article gives an overview of female reproductive biology and the role of large-scale data analysis and -omics techniques in the diagnosis, prognosis, and management of female reproductive disorders. Also, the role of machine learning approaches for predictive models in prevention and management has been discussed. Personalizing maternity care by use of mobile apps and wearable devices in continuous monitoring of female (fertility-related) health and prediction of any early complications to provide personalized intervention solutions. These technologies should be implemented in the national healthcare planning systems by utilizing effective clinical decision-support tools and new educational models and machine-learning approaches along with appropriate resource allocation.

This series has focused on the role of eDiagnostics and precision medicine in endocrinology. Recent technological advances in -omics and wearable devices have transformed the rate of clinically relevant data production. Generation of data is no longer a bottleneck, but the analysis and interpretation of data into actionable information, allowing earlier diagnosis and treatment options. Sensor technologies allows tracking of heart rate, blood glucose, sleep, breath, voice etc. opening avenues for new digital biomarkers discovery. Clinical decision support systems will need high-performance algorithms to make use of big data, including the diversity it presents. Additionally, new protocols for sharing information as well as integrating patient data must be integrated in the clinical decision support systems for improved diagnosis, therapy assessment and prevention. Healthcare professionals will require training to implement and incorporate these applications into their everyday practice. Personalized preventive medicine thus can be facilitated by estimating an individual’s disease risk and clinical response of a disease to a particular therapeutic option and applying personalized management accordingly.

## Author contributions

SK conceived the special issue. SK and AJ wrote a review article and wrote the editorial. All authors approved the final version.
